# 15 Years of Inclusive Employment in a Down Syndrome Clinic

**DOI:** 10.3233/WOR-240080

**Published:** 2024-11-08

**Authors:** Sydney S. Reynders, Angela M. Lombardo, Emily J. Davidson, Jennifer L. Guan, Katherine G. Pawlowski, Nathan Z. Simons, Nicole T. Baumer

**Affiliations:** a Division of Developmental Medicine, Boston Children’s Hospital, Boston, MA, USA; b Department of Neurology, Boston Children’s Hospital, Boston, MA, USA; c Harvard Medical School, Boston, MA, USA

**Keywords:** Intellectual disability, developmental disabilities, healthcare, work, mentoring, supported employment

## Abstract

**BACKGROUND:**

Adults with intellectual disabilities have high rates of unemployment and underemployment. Despite benefits to employers and employees, some groups may be hesitant to implement inclusive employment programs due to lack of knowledge, absence of well-defined strategies, and limited exposure to successful examples.

**OBJECTIVE:**

To address this gap, the Down Syndrome Program (DSP) in a New England tertiary pediatric hospital established an inclusive employment program that supports and trains young adults with Down syndrome in the development of foundational job skills within a hospital-based clinic.

**METHODS:**

This case study examines strategies and lessons learned from the employment program’s implementation and evolution.

**RESULTS:**

Successful implementation required iterative, tailored approaches to meet diverse needs.

**CONCLUSION:**

The DSP developed a framework and collection of best practices for other organizations to adopt for successful employment of individuals with disabilities under an inclusive employment model.

## 1 Introduction

Down syndrome (DS) is characterized by intellectual disability (ID) with variable profiles [1, 2] including impairments in aspects of learning and memory [3]. Health outcomes have improved with therapies targeting language, adaptive skills, independence, and quality of life [4]. Despite advances, individuals with DS face challenges gaining and retaining employment, in part due to negative perceptions about inclusion of individuals with disabilities in the workplace [5, 6]. Estimates show that only 20% of adults with DS in the United States are employed [7], similar to rates for individuals with disabilities (21.3%), but extremely low compared to 65.4% of people without disabilities [8].

For individuals with disabilities, employment improves quality of life, self-confidence, self-advocacy skills, and social connectedness [9, 10]. For individuals with DS, employment promotes independence and enhances quality of life [11]. Inclusion of individuals with DS in the workplace can enhance organizational health [10, 11], demonstrating the benefit of inclusive employment efforts to organizations [12]. While prior research has identified a need for attitudinal and structural changes in the workplace to improve inclusion [13], very few healthcare institutions have effectively implemented these changes [19, 20], underscoring the need for replicable inclusive employment models.

The purpose of this paper is to 1) describe the development and implementation of an inclusive employment program in a clinical workplace, 2) outline lessons learned over 15 years and best training practices, and 3) provide resources and framework for other organizations looking to adopt an inclusive employment model.

## 2 Case study

The Down Syndrome Program (DSP) is a specialized clinic in a tertiary hospital offering multidisciplinary care to over 500 children and adolescents with DS each year. In 2009, the DSP hired an individual with DS for a two-year, part-time position. Subsequently, the DSP dedicated resources to establishing a training and inclusive employment program to support adults with DS. Since its inception, the employment program has hired six employees with DS between 2009–2024 and created two permanent positions within the DSP.

An integral aim was to strengthen foundational job skills in a clinical setting to increase employment opportunities upon completion. In addition, the employment program sought to demonstrate employment possibilities to families receiving care in the DSP clinic. By employing individuals with DS and embracing their unique value and skills, the DSP gained crucial knowledge of how best to support employees with DS.

### 2.1 Development and iteration

From its inception, the employment program consisted of a paid, two-year, part-time job training position for individuals with DS. The design allowed individuals with DS to gain clinical skills and exposure working as patient liaisons. The employment program hosted both team and department-wide training sessions to educate colleagues on learning and communication profiles in DS, equipping colleagues with an understanding of how to effectively support differing abilities in the workplace.

Consistent with a continuous improvement model [14], individuals with DS hired under the employment program had an essential role in its development and improvement. As the employment program evolved, the DSP recognized the immense contributions of participants, leading to the development of two permanent positions within the DSP for individuals who successfully completed the training and were interested in remaining employed in healthcare: a full-time patient liaison and a part-time research assistant. Positions were designed in collaboration with these individuals to align with their strengths and interests, ensuring that they could effectively contribute and be supported. This established a precedent for employment of individuals with disabilities in the hospital and marked the expansion of the employment program from a training initiative to a sustainable model of inclusive employment.

### 2.2 Hiring process

Individuals applying to the employment program followed the hospital application process: online application, submission of a letter of interest and resume, and interviews conducted by program supervisors. Most applicants were between 22 and 25 years old, had completed post-secondary education, and were not actively enrolled in an academic program or other paid employment. The employment program sought applicants with little to modest work experience and an eagerness to learn job skills unique to healthcare. Final candidates observed clinic sessions and met team members. Consideration was given to an applicant’s ability to behaviorally navigate and participate in a fluctuating clinical setting and to the employment program’s potential impact on the applicant’s employment trajectory. Applicants still completing secondary education were encouraged to apply when older. Those not hired were provided resources and local recommendations for other suitable employment opportunities.

Once hired, participants completed hospital onboarding and orientation, familiarized themselves with team members and the hospital environment, and received hands-on working experience. Participants completed annual trainings required of all hospital employees.

### 2.3 Job skills

**Fig. 1:**
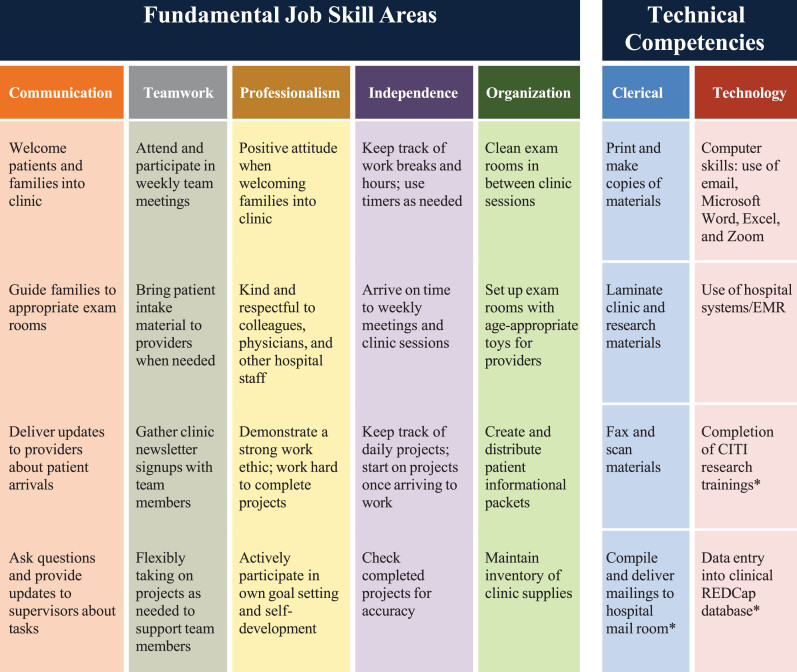
Participant responsibilities and job skill aims chart. *Note.* Responsibilities and skills fell into seven main categories, with example job tasks and goals listed for each area. ^*^denotes additional job skills as part of the regular activities of the current permanent employees with DS.

To support future employment, the employment program focused on seven categories of fundamental job skills: communication, teamwork, professionalism, independence, organization, clerical skills, and technology. Participants served as patient liaisons: touchpoints between families and medical staff. Figure 1 depicts participant responsibilities, categorized by skills and competencies reinforced.

### 2.4 Supervision and natural coaching

The DSP Senior Program Coordinator, central to patient education and care, and a clinical research assistant served as primary supervisors to employees in the employment program. They provided daily, personalized mentorship and weekly check-in meetings. Supervision utilized strengths-based coaching, which leverages existing abilities in a supportive environment [15, 21] and is shown to positively impact confidence, self-esteem, and activity participation in adolescents with autism [15]. Recognizing that individuals with DS possess relative strengths in observational and visual learning [3], supervisors adopted a hands-on approach when teaching new tasks. Participants observed supervisors perform a task and then worked either alongside supervisors to complete the task together or independently under supervision. Incorporating experiential learning, shown to facilitate immediate application of knowledge to real-world experiences [16], participants actively reflected when performing tasks and received real-time teaching and continuous feedback from supervisors. The permanent hiring of two individuals with DS also created peer mentoring support within the employment program, known to positively impact learning outcomes [17, 18]. These role models demonstrated tasks and provided guidance. By prioritizing exploration of strengths and interests, the employment program cultivated an enthusiasm for learning.

**Fig. 2:**
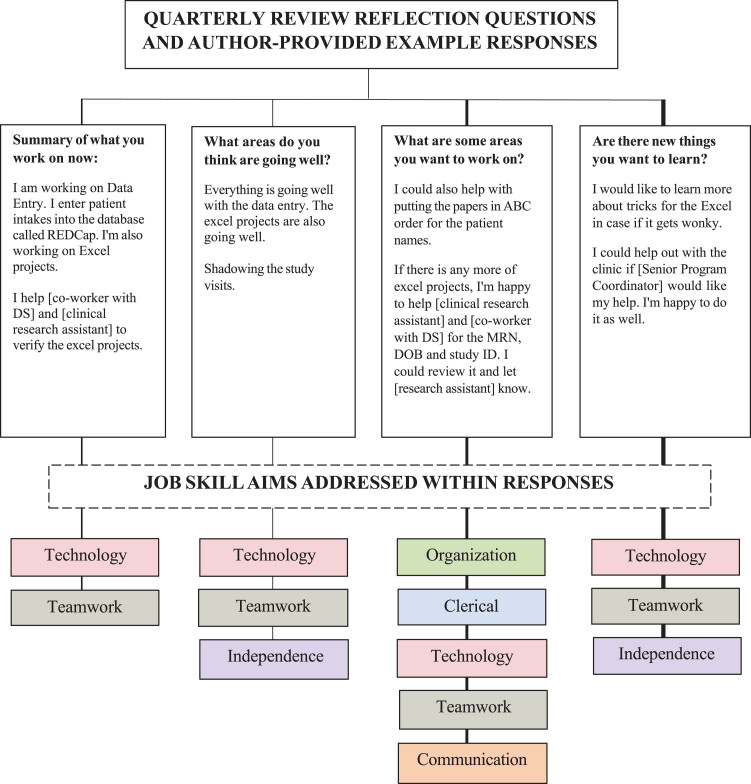
Quarterly review reflection questions and author-provided example responses. *Note.* Author is a self-advocate and previous participant in the DSP employment program who now works as a current DSP employee. Example responses addressed several job skill aims presented in Figure 1; corresponding job skill aims are listed below each response.

Participants took ownership of their growth through quarterly reviews with supervisors, an opportunity to engage in discussions and individualized goal setting. Prior to review sessions, participants identified accomplishments, areas for improvement, and new goals, fostering a collaboration where participants actively shaped their work goals. Review sessions enabled supervisors to track and adjust responsibilities and facilitate growth. Figure 2 illustrates quarterly review questions and author-participant example responses.

## 3 Discussion

### 3.1 Lessons learned

Individuals with DS have diverse skills and unique needs, necessitating customized, iterative approaches to accommodate personal strengths and challenges in the workplace. Some participants faced challenges in recalling verbal instructions and self-monitoring behavior while others had difficulties with time management. Challenges were addressed using a tailored approach, including visual supports and behavioral prompts. There was a consistent relative strength of visual learning, aligning with existing knowledge about cognitive profiles in DS [3]. Notable advances were observed in independence and personal responsibility.

An initial challenge of the employment program was the lack of familiarity amongst colleagues regarding learning styles of individuals with DS. Conducting training sessions to increase understanding of how to effectively collaborate with co-workers with DS was integral to the employment program’s long-term success. Educating colleagues within the department about the unique needs of individuals with DS provided ongoing advocacy for a broader commitment to inclusion, diversity, and equal opportunities within the organization.

Through spontaneous feedback, families revealed their inspiration at witnessing adults with DS contribute within the clinic and serve as role models for their own children with DS, voicing appreciation for the supportive working environment. Colleagues took pride in their department’s commitment to inclusive employment, acknowledged personal growth in effective collaboration, expressed gratitude for skills gained in working with diverse learning populations, and shared their deepened understanding of differing disabilities within their personal circles. Employees with DS expressed appreciation for skills learned, meaningful relationships formed with co-workers, and joy experienced in working with children with DS.

### 3.2 Recommendations and prescription of best practices

**Fig. 3:**
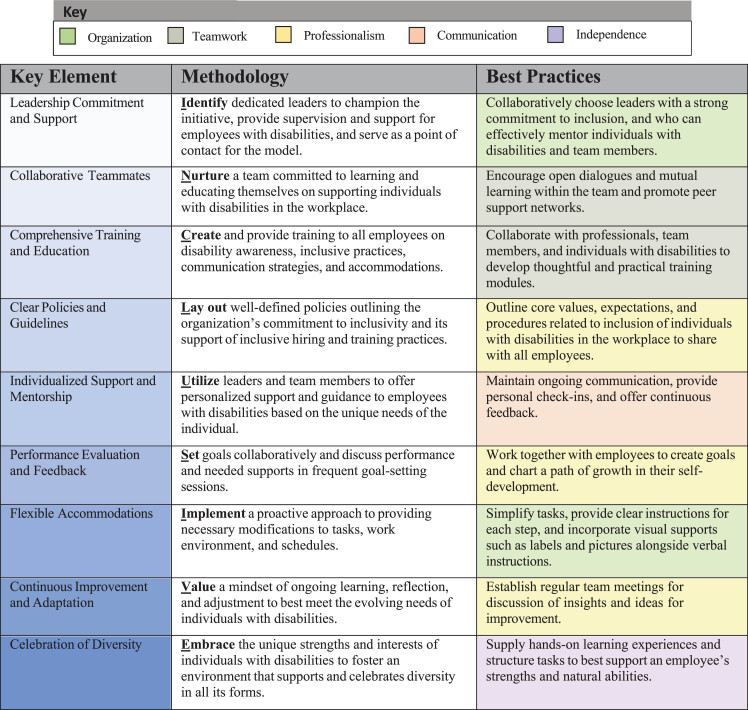
INCLUSIVE framework for implementation of an inclusive employment model. *Note.* Nine key elements were identified for the INCLUSIVE framework and paired with methodology and best practices for implementation. Best practices for each key element are color categorized according to fundamental job skill aims represented in Figure 1. Best practices cover the following areas: Organization, Teamwork, Professionalism, Communication, and Independence.

Successful implementation of the DSP’s employment program required the following key elements: 1) Leadership commitment and support; 2) Collaborative teammates; 3) Comprehensive training and education; 4) Clear policies and guidelines; 5) Individualized support and mentorship; 6) Performance evaluation and feedback; 7) Flexible accommodations; 8) Continuous improvement and adaptation; and 9) Celebration of diversity. Figure 3 summarizes elements within the INCLUSIVE framework for other organizations to follow. Best practices for supporting employees are described in Fig. 3, classified according to job skill.

### 3.3 Limitations

We did not collect detailed applicant demographic information, so cannot assess whether some individuals may not have access to our program. Behavioral and language limitations may also hinder participation. Staff availability and institutional support may prevent replication.

## 4 Summary

To address high unemployment rates among adults with disabilities, the DSP developed an inclusive employment program within a healthcare environment and found that successful implementation relied on iterative, tailored approaches to meet diverse needs. The DSP created the INCLUSIVE framework for other organizations, to facilitate hiring individuals with disabilities.

## Ethical approval

Not applicable.

## Informed consent

Not applicable.

## Conflict of interest

The authors declare that they have no conflicts of interest.
